# Extracellular signal-regulated kinases associate with and phosphorylate DHPS to promote cell proliferation

**DOI:** 10.1038/s41389-020-00271-1

**Published:** 2020-09-28

**Authors:** Chao Wang, Zhen Chen, Litong Nie, Mengfan Tang, Xu Feng, Dan Su, Huimin Zhang, Yun Xiong, Jeong-Min Park, Junjie Chen

**Affiliations:** Department of Experimental Radiation Oncology, The University of Texas MD Anderson Cancer Center, Houston, TX 77030 USA

**Keywords:** Lung cancer, Phosphorylation

## Abstract

The ERK1/2 pathway is one of the most commonly dysregulated pathways in human cancers and controls many vital cellular processes. Although many ERK1/2 kinase substrates have been identified, the diversity of ERK1/2 mediated processes suggests the existence of additional targets. Here, we identified Deoxyhypusine synthase (DHPS), an essential hypusination enzyme regulating protein translation, as a major and direct-binding protein of ERK1/2. Further experiments showed that ERK1/2 phosphorylate DHPS at Ser-233 site. The Ser-233 phosphorylation of DHPS by ERK1/2 is important for its function in cell proliferation. Moreover, we found that higher DHPS expression correlated with poor prognosis in lung adenocarcinoma and increased resistance to inhibitors of the ERK1/2 pathway. In summary, our results suggest that ERK1/2-mediated DHPS phosphorylation is an important mechanism that underlies protein translation and that DHPS expression is a potent biomarker of response to therapies targeting ERK1/2-pathway.

## Introduction

The extracellular signal-regulated kinase 1/2 (ERK1/2) belongs to an essential signaling pathway that regulates various stimuli-induced cellular processes, including cell proliferation, differentiation, and survival^1–4^. ERK1/2 is often activated by signals initiated at cell membrane, which involve membrane-associated receptors, such as receptor Tyr kinases^5^. These receptors instigate signal transduction by recruiting adaptor proteins and activating Ras-GTPases, which induce the activation of protein kinases Rafs (Raf-1, B-Raf, and A-Raf)^6^. The activated Rafs phosphorylate the MAPKKs (MEK1/2), which further phosphorylate and activate ERK1/2^7^. Activated ERK1/2 has a wide range of substrates, including transcription factors (Elk1, c-Fos, and c-Jun) and other kinases (RSK, MNKs-MAPK-interacting protein kinase, and mitogen- and stress-activated protein kinase)^2,8–13^. These substrates of ERK1/2 are important executers of the ERK1/2-dependent signaling cascade and participate in the regulation of a variety of cellular processes^2^. Moreover, abnormal regulation of ERK1/2 pathway has been reported in many different types of cancers, and drugs targeting this pathway are being used or tested for cancer treatment^2,8,14–16^.

Although many substrates of ERK1/2 have been identified to date, the diverse roles of ERK1/2 signaling within the cell suggest that there may be more yet to be identified substrates of these key kinases^17^. In addition, despite the success of inhibitors targeting the ERK1/2 pathway, their efficacies are compromised by the emerging drug resistance^18,19^. Identification of more ERK1/2 substrates may shed light on these questions.

Deoxyhypusine synthase (DHPS)-dependent hypusination is an important posttranslational process that only exists in eukaryotic cells^20–23^. This unique process is a catalytic cascade that is directed by DHPS and deoxyhypusine hydroxylase and ultimately generates hypusinated eIF5A^21,22^. Hypusinated eIF5A is essential for general translation elongation and termination and is recognized as a critical regulator of cell growth and tumor development^24–26^. A recent study in mouse revealed that DHPS depletion impaired β cell proliferation and induced overt diabetes, suggesting a possible role of hypusination in metabolism^27^. Another recent study associated reduced DHPS activity with neurodevelopment disorder^28^. These studies highlight the critical functions of hypusination process. In these biochemical reactions, DHPS catalyzes the rate-limiting step, which is the use of polyamine spermidine as the substrate to synthesize amino acid hypusine^20,23^. Although the role of DHPS in hypusination process has been well studied and DHPS itself is recognized as a potential target for intervention in cell proliferation^24,27,29,30^, how the activity of DHPS is regulated remains largely unknown.

To look for more ERK1/2 related proteins, we performed a proteomics study of ERK2 and identified DHPS as a major ERK1/2-associated protein. Conversely, ERK1/2 was also identified as a major DHPS-binding protein, suggesting a close functional relationship between these proteins. We further demonstrated that DHPS binds directly to ERK1/2 through its ERK-binding-motif (EBM) region. In addition, we mapped the Ser-233 site of DHPS as the major ERK phosphorylation site on DHPS. While the expression of wild-type DHPS or its phospho-mimetic mutant S233D was able to rescue the impaired cell proliferation induced by DHPS knockdown, the expression of phosphorylation-deficient mutant DHPS-S233A was partially defective. Together, our results not only reveal a novel regulation of DHPS but also a new function of the ERK1/2 signaling pathway in the control of the DHPS-eIF5a axis, which is important for protein translation.

## Results

### DHPS is a major binding protein of ERK1/2

To search for more binding partners of ERK1/2, we performed tandem affinity purification (TAP), coupled with mass spectrometry (MS) analysis, as previously described^31^. We performed this study with ERK2 (also called MAPK1), as ERK1 (also called MAPK3) and ERK2 share a protein sequence with 84% identity^1^. In addition, we used another closely related MAPK kinase ERK5 as a control^1^.

**Fig. 1:**
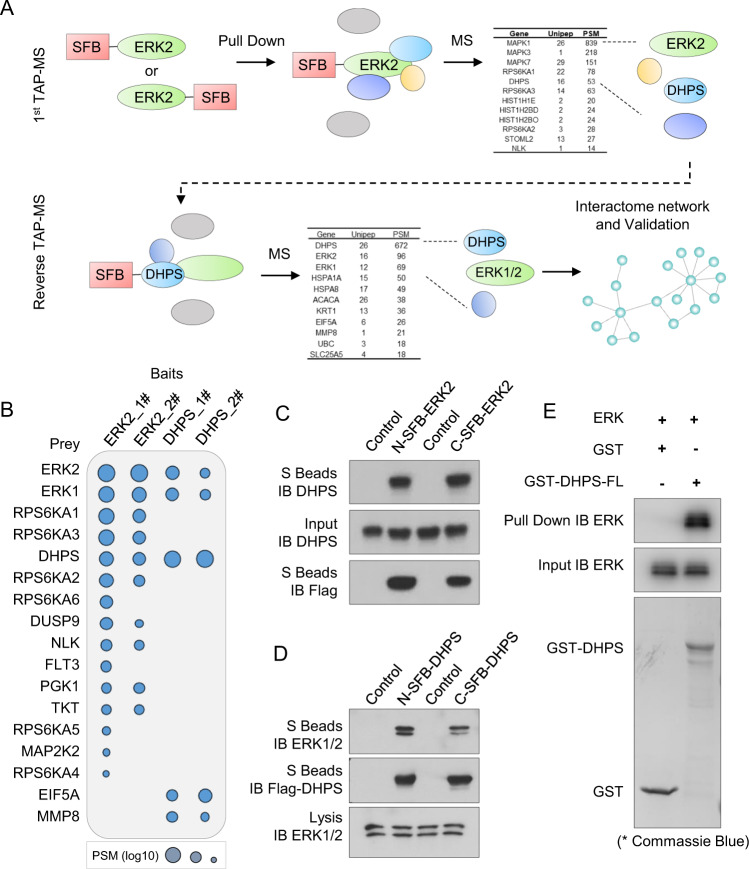
Reciprocal TAP-MS Analysis Uncovered DHPS and ERK2 Interactome. **a** Workflow of the protein interactome study using the TAP-MS approach. Constructs encoding N- or C-terminal SFB-tagged ERK2 were expressed in HEK293T cells. Cell lysates underwent two-step affinity purification using streptavidin beads and S-protein agarose beads. The samples were then analyzed by MS, and the identified peptides were filtered using HCIP analysis. The potential ERK2 interactor DHPS was fused to N- or C-terminal SFB tag and underwent the same TAP-MS analysis. **b** Visualization of the reciprocal TAP-MS results of ERK and DHPS. The HCIP list for each bait is displayed as a dot plot. The PSMs (log10) of the identified proteins are determined by the size of the circles. **c** ERK2 interacts with DHPS. N- or C-terminal SFB-tagged ERK2 was expressed in HEK293T cells. Cell lysates were pulled down with S protein beads, and the indicated proteins were detected by Western blotting. Data represent a representative experiment from three independent experiments. **d** Association between DHPS and ERK1/2. N- or C-terminal SFB-tagged DHPS were expressed in HEK293T cells. Cell lysates were pulled down with S protein beads, and the indicated proteins were detected by Western blotting. Data represent a representative experiment from three independent experiments. **e** In vitro interaction between DHPS and ERK. ERK protein was incubated with GST protein or GST-DHPS protein. The pull-down assays were performed with glutathione agarose, and the indicated proteins were detected by Western blotting or Coomassie Blue staining.

As shown in Fig. 1a, we first generated HEK293T-derivative cell lines stably expressing N-terminal SFB-tagged or C-terminal SFB-tagged ERK2. Cell lysates were then extracted, followed by TAP purification. The enriched proteins were digested by Trypsin and analyzed by MS; they were then subjected to a SAINT analysis to obtain a list of highly confidential interaction proteins (HCIP). As shown in Supplementary Table 1 and Supplementary Fig. 1A, the ERK2-interacting protein list, but not the control ERK5-interacting protein list, comprised known ERK1/2 interactors, such as RPS6KA1, RPS6KA3 and DUSP9. We also identified a previously unknown ERK2 interactor, DHPS.

To further confirm these results, we performed reciprocal TAP-MS interactome study of DHPS. As shown in Supplementary Table 1 and Supplementary Fig. 1B, the TAP-MS results suggest that ERK1/2 is a robust interactor of DHPS. The top interactors of ERK2 and DHPS are listed in Fig. 1b, suggesting that DHPS is a major binding protein of ERK2.

### DHPS directly binds to ERK1/2 through its EBM

To further confirm the interaction between DHPS and ERK1/2, we performed transient transfection and co-immunoprecipitation (co-IP) experiments. As shown in Fig. 1c and Supplementary Fig. 1C, SFB-tagged ERK2 protein interacted with endogenous DHPS, while the control protein ERK5, which showed close similarity with ERK1/2^4^, failed to do so. We also performed reciprocal co-IP assays, which showed that SFB-tagged DHPS could readily bind to endogenous ERK1/2 (Fig. 1d). To further elucidate whether the interaction between DHPS and ERK1/2 is regulated by ERK1/2 phosphorylation or activation, we performed co-IP experiments using mock-treated cells or cells that had been treated with phorbol 12-myristate 13-acetate (PMA) that could activate the ERK1/2 pathway^32^. As shown in Supplementary Fig. 1D, while ERK1/2 were activated and phosphorylated after PMA treatment, the binding between DHPS and ERK1/2 were not affected, indicating that the interaction between DHPS and ERK1/2 does not depend on ERK1/2 phosphorylation or activation.

To determine whether the interaction between DHPS and ERK was direct, we performed in vitro pull-down assays with GST-DHPS and ERK protein. As shown in Fig. 1e, GST-DHPS, but not the GST control protein, could bind to ERK, suggesting a direct binding of DHPS with ERK.

**Fig. 2:**
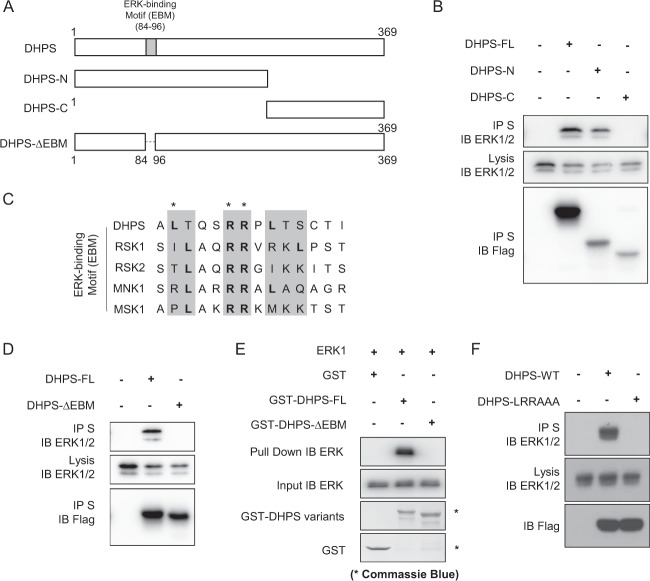
DHPS Interacts with ERK1/2 through Its EBM. **a** Schematic illustration of the DHPS deletion variants used below. **b** Interaction between the N-terminus of DHPS and ERK1/2. SFB-tagged full length (FL) or N- or C-terminus of DHPS was expressed in HEK293T cells. Cell lysates were pulled down with S protein beads, and the indicated proteins were detected by Western blotting. Data represent a representative experiment from three independent experiments. **c** Sequence alignment between DHPS and other ERK-binding proteins indicate a possible EBM. The basic residues are marked with asterisks. **d** Deletion of EBM in DHPS abolishes the binding between DHPS and ERK1/2. SFB-tagged full length (FL) or ∆EBM of DHPS was expressed in HEK293T cells. Cell lysates were pulled down with S protein beads, and the indicated protein was detected by Western blotting. Data represent a representative experiment from three independent experiments. **e** In vitro interaction between DHPS-FL/∆EBM and ERK. Purified ERK protein was incubated with GST, GST-DHPS-FL protein, or GST-DHPS-∆EBM protein. The pull-down assay was performed with glutathione agarose, and the indicated proteins were detected by Western blotting or Coomassie Blue staining. **f** Mutations of negatively charged residues in EBM of DHPS abolish the binding between DHPS and ERK1/2. SFB-tagged WT or LRRAAA variant of DHPS were expressed in HEK293T cells. Cell lysates were pulled down with S protein beads, and the indicated proteins were detected by Western blotting. Data represent a representative experiment from three independent experiments.

To further identify the region of DHPS that is required for its interaction with ERK1/2, we first generated DHPS N-terminal and C-terminal truncation mutants (Fig. 2a). As shown in Fig. 2b, co-IP experiments indicated that DHPS interacts with ERK1/2 with its N-terminus. By comparing the N-terminal sequence of DHPS protein with the sequences of several known ERK1/2 binding proteins (i.e., RSK1/2, MNK1, and MSK1), we identified a possible EBM at the N-terminus of DHPS (Fig. 2c and Supplementary Fig. 2A, B), which comprises several basic residues (LXXRRXX). This possible EBM is also highly conserved among many species (Supplementary Fig. 2A). We then developed the DHPS-∆EBM mutant in which the EBM was deleted. The following co-IP experiment showed that the DHPS-∆EBM mutant was unable to co-precipitate with ERK1/2 (Fig. 2d). Moreover, in vitro pull-down assays also showed that GST-DHPS-∆EBM mutant failed to bind to ERK (Fig. 2e).

To determine whether the basic residues within EBM are essential for the binding between DHPS and ERK1/2, we generated DHPS-EBM-RRAA (double mutation) and DHPS-EBM-LRRAAA (triple mutation) variants and performed co-IP experiments. As shown in Supplementary Fig. 2C and Fig. 2f, the RRAA mutant dramatically decreased the binding between DHPS and ERK1/2, while the LRRAAA mutant abolished this interaction. All of the above results suggest that DHPS directly interacts with ERK1/2 through its EBM.

### DHPS is phosphorylated by ERK1/2 at Ser-233

**Fig. 3:**
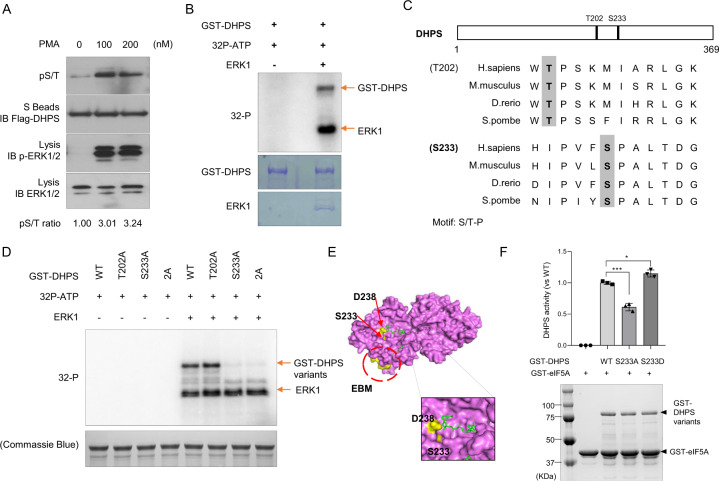
Identification of Ser-233 in DHPS as an ERK Phosphorylation Site. **a** Western blotting of exogenous DHPS phosphorylation in HEK293A cells. HEK293A cells expressing SFB-tagged DHPS were exposed to DMSO or different dosages of PMA for 30 min. Cell lysates were pulled down with S protein beads, and the indicated proteins were detected by Western blotting. Relative levels of pS/T signals after normalization to the flag signals were shown. Data represent a representative experiment from three independent experiments. **b** In vitro kinase assay of DHPS. Purified GST or GST-DHPS was incubated with or without ERK. γ-^32^P signals were detected by PhosphorImager. The proteins were detected by Coomassie Blue staining. Arrows mark the radiative signals of GST-DHPS phosphorylation and ERK auto-phosphorylation. **c** Sequence alignment indicates two possible ERK phosphorylation sites (S/T-P) that are highly conserved from yeast to humans. **d** In vitro kinase assay of DHPS variants. Purified GST-DHPS-WT or variants (T202A, S233A, 2A/T202A-S233A) were incubated with or without ERK. γ-^32^P signals were detected by PhosphorImager. The proteins were detected by Coomassie Blue staining. Arrows marked the radiative signals of GST-DHPS phosphorylation and ERK self-phosphorylation. Data represent a representative experiment from three independent experiments. **e** Surface of the human DHPS-dimer crystal structure (PDB: 1ROZ). The substrates are marked as green (upper: NADPH, lower: spermidine). Ser-233 (S233) is marked with yellow and is located in the NADPH binding pocket. The other essential amino acid, Asp-238 (D238), is also marked with yellow. The EBM described in this study is circled and marked with yellow. A close-up of the surface, including the Ser-233 site, is shown in the right panel. **f** Comparison of enzymatic activities of DHPS variants. The enzymatic activity assays were performed with purified GST-eIF5A (1 μg proteins in each sample) and different GST-DHPS variants (0.2 μg proteins in each sample). The proteins were detected by Coomassie Blue staining. Arrows mark the proteins of GST-DHPS variants and GST-eIF5A. The activities of the mutant enzymes are expressed as percent of the wild-type enzyme in each group. Results were presented as means ± S.D. from three independent experiments. ***, *p* < 0.001. *, *p* < 0.05. *P* values were obtained using Student’s *t* test.

Since DHPS is a robust ERK1/2 interacting protein, we determined whether DHPS is regulated by ERK1/2. We treated the cells with different doses of PMA to activate ERK1/2 and detected the phosphorylation status of overexpressed DHPS. As shown in Fig. 3a, while ERK1/2 was activated after PMA treatment, the phosphorylation of DHPS was also elevated, as detected by the anti-phospho-Ser/Thr antibody.

To determine whether DHPS is directly phosphorylated by ERK1/2, we performed in vitro kinase assays with purified MBP-DHPS protein and ERK protein. As shown in Fig. 3b, GST-DHPS protein could be phosphorylated by ERK, suggesting that DHPS is a substrate of ERK1/2.

To determine the ERK1/2 phosphorylation sites on DHPS protein, we evaluated the DHPS protein sequence and identified two putative ERK1/2 phosphorylation sites (T202 and S233, the S/T-P motifs) (Fig. 3c). We then generated and purified DHPS variants (DHPS-T202A, DHSP-S233A, and DHPS-T202A/S233A) and performed in vitro kinase assays using these mutant proteins. As shown in Fig. 3d, while wild-type and the DHPS-T202A mutant were phosphorylated by ERK in vitro, the DHPS-S233A and DHPS-T202A/S233A (2A) mutants failed to do so, suggesting that Ser-233 is the major ERK1/2 phosphorylation site. As identified through the observation of the reported DHPS crystal structure^33–35^, Ser-233 locates in the substrate NADPH binding groove of DHPS (Fig. 3e), which suggests that phosphorylation of this site may influence DHPS enzymatic activity. We further performed in vitro enzymatic activity assays for DHPS. As shown in Fig. 3f, DHPS-S233A exhibited decreased activity, while DHPS-S233D had slightly increased activity, suggesting that phosphorylation of DHPS is essential for its optimal activity.

### Ser-233 phosphorylation of DHPS is important for its function in cell proliferation

**Fig. 4:**
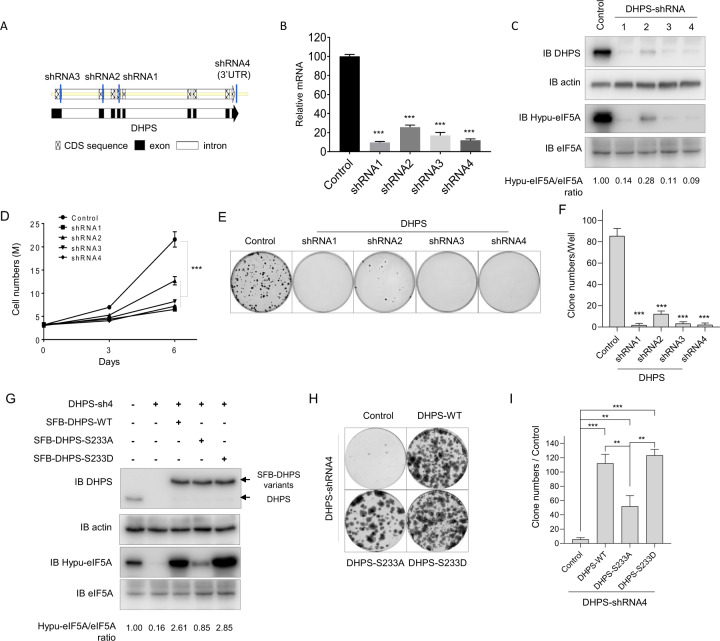
Phosphorylation of Ser-233 Influences Cell Proliferation. **a** Schematic illustration of the DHPS shRNAs used in this study. CDS (coding DNA sequence), exon, intron are marked as indicated. **b** qPCR was performed to check the knockdown efficiency of different shRNAs. HEK293A cells were infected with the indicated shRNAs. Total RNA from different groups were extracted, and DHPS mRNA levels were checked by qPCR. Results were presented as means ± S.D. from three independent experiments. ***, *p* < 0.001. *P* values were obtained using Student’s *t* test. **c** Western blotting was performed to check the knockdown efficiency of different shRNAs. The cell lysates from the same groups as in (**b**) were detected with the indicated antibodies. Relative levels of Hypusinated eIF5A (Hypu-eIF5A) to total eIF5A after normalization to the control were shown. Data represent a representative experiment from three independent experiments. **d** Cell growth curve experiments were performed using the same cells as in (**b**). Cells were plated in 6-well plates, and cell numbers were counted every 3 days. Results were presented as means ± S.D. from three independent experiments. **, *p* < 0.01. *P* values were obtained using Student’s *t* test between the control group and shRNA groups. **e** Clonogenic formation experiments were performed using the same cells as those in (**b**). Cells were plated at 6-well plates at 150/well. After 10 days of incubation, cells were stained with crystal violet and counted. **f** Statistical analysis of the clonogenic formation experiments in (**e**). The results are presented as means ± S.D. from three independent experiments. ***, *p* < 0.001. *P* values were obtained using Student’s *t* test between the control group and shRNA groups. **g** Western blotting were performed with the indicated reconstituted cells. HEK293A cells ectopically expressing DHPS-WT/DHPS-S233A/DHPS-S233D were established. These cells were then infected with DHPS-shRNA4. The cell lysates were detected with the indicated antibodies. Relative levels of Hypusinated eIF5A (Hypu-eIF5A) to total eIF5A after normalization to the control were shown. Data represent a representative experiment from three independent experiments. **h** Clonogenic formation experiments were performed with the indicated reconstituted cells. HEK293A cells ectopically expressing DHPS-WT/DHPS-S233A/DHPS-S233D were established. These cells were then infected with DHPS-shRNA4. Cells were plated in 6-well plates at 150/well. After 14 days of incubation, cells were stained with crystal violet and counted. **i** Statistical analysis of the clonogenic formation experiments in (**e**). Results are presented as means ± S.D. from three independent experiments. ***, *p* < 0.001. *P* values were obtained using Student’s *t* test between different groups.

DHPS-dependent posttranslational modification of eIF5A1/2 via hypusination was reported to be vital for cell viability, cell proliferation, and tumor metastasis^21,24^. To validate the function of DHPS in cell proliferation, we knocked down DHPS with several different DHPS shRNAs (Fig. 4a). DHPS levels were efficiently knocked down, which was confirmed at both mRNA and protein levels (Fig. 4b, c). It is also of note that the hypusination levels of eIF5A were decreased dramatically in DHPS knockdown cells (Fig. 4c). DHPS knockdown cells grew much more slowly than control WT cells (Fig. 4d). In addition, colony formation assays confirmed that DHPS knockdown cells showed dramatically decreased colony formation ability (Fig. 4e, f). Indeed, this reduction in cell proliferation or colony formation was correlated well with the depletion of DHPS expression, since cells transfected with shRNA2, which showed residual DHPS expression (Fig. 4b, c), also displayed intermediate phenotypes in cell proliferation and colony formation assays (Fig. 4d–f). These results confirmed the critical role of DHPS in cell proliferation.

To further define the physiological function of DHPS Ser-233 phosphorylation by ERK, we first generated 293 A cells ectopically expressing WT DHPS, phosphorylation-deficient DHPS-S233A mutant, or phospho-mimetic DHPS-S233D mutant. We then knocked down DHPS in these cells and compared their colony formation abilities. As expected, DHPS knockdown led to decreased level of hypusinated eIF5A, while overexpression of DHPS-WT/S233D efficiently rescued the hypusination levels of eIF5A whereas overexpression of DHPS-S233A could only partially rescue eIF5A hypusination (Fig. 4g). Consistently, while WT DHPS and DHPS-S233D mutant efficiently rescued the colony formation ability after DHPS knockdown, the DHPS-S233A mutant displayed significantly diminished ability to rescue colony formation (Fig. 4h, i). Together, these data suggest that ERK-mediated Ser-233 phosphorylation of DHPS is important for its function in promoting cell proliferation through regulating its enzyme activity.

### Higher expression levels of DHPS correlate with poor prognosis in lung adenocarcinoma and resistance to inhibition of the ERK1/2 pathway

**Fig. 5:**
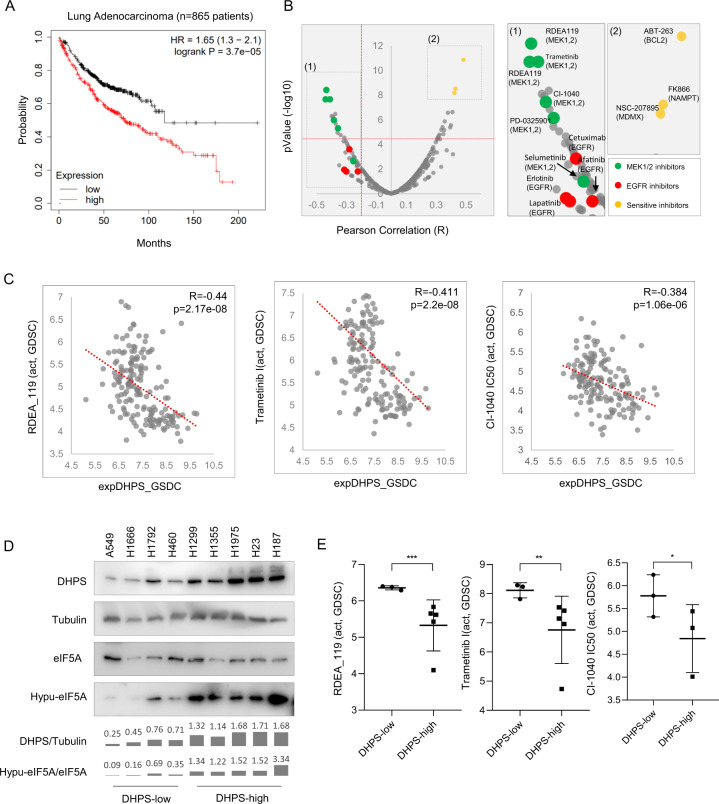
Higher Expression Levels of DHPS Are Correlated with Poor Prognosis in Lung Adenocarcinoma and Resistance to Inhibition of the ERK1/2 Pathway. **a** Kaplan–Meier plot of lung adenocarcinoma patients (*n* = 865). Data are from Kaplan–Meier Plotter (http://kmplot.com) and DHPS probe is 211558_s_at. The *p* value is listed in the panel. **b** Correlation analysis between expression levels of DHPS and the drug activity data in lung cancer cell lines. Pearson correlations between expression levels of DHPS and GDSC drug activities were calculated and plotted. The MEK1/2 inhibitors are marked with green, the EGFR inhibitors are marked with red, and the positive correlated drugs are marked with yellow. –log10[IC50M] was used to mark the drug activity. **c** Higher expression levels of DHPS correlate with increased sensitivity to multiple MEK1/2 inhibitors. The top negative correlated drugs from (**b**) were plotted. The Pearson correlation *R* and *p* values are labeled in the panels. **d** Western blotting were performed with the indicated lung cancer cell lysates. Relative levels of DHPS after normalization to those of tubulin were shown with values and bar plots. Relative levels of Hypusinated eIF5A (Hypu-eIF5A) after normalization to total eIF5A were shown with values and bar plots. Data represent a representative experiment from three independent experiments. **e** Comparison plot for indicated GDSC drug activities of the lung cancer cell lines analyzed in (**d**). –log10[IC50M] was used to mark the drug activity. ***, *p* < 0.001. **, *p* < 0.01, *, *p* < 0.05. *P* values were obtained using One sample *t*-test between different groups.

DHPS induced hypusination was found to correlate with prognosis and metastasis in several cancer types, such as esophageal squamous cell carcinoma and pancreatic cancer^30,36^. In this study, we found that higher DHPS expression levels were associated with poor prognosis in lung adenocarcinoma (Fig. 5a), which suggests that DHPS can be used as a marker for cancer prognosis.

To determine whether drug sensitivity was correlated with DHPS expression levels, we analyzed the Genomics of Drug Sensitivity in Cancer datasets of lung cancer cells^37,38^. We compared the expression levels of DHPS with responses to more than 297 drugs across 169 lung cancer cell lines (Supplementary Table 2); DHPS expression correlated negatively with cellular sensitivity to inhibitors of EGFR, BRAF, or MEK1/2 (Fig. 5b, c and Supplementary Fig. 3), indicating that high DHPS expression renders these cells and tumors more resistant to kinase inhibitors that target this pathway. We further tested several lung cancer cell lines. As shown in Fig. 5d, e, the lung cancer cell lines with higher DHPS protein levels showed higher hypusination levels of eIF5A and are more resistant to these inhibitors. On the other hand, DHPS expression appeared to correlate positively with sensitivity to other agents, such as NAMPT, BCL2, and MDMX inhibitors (Fig. 5b), indicating that DHPS level can be used as a predictive biomarker for response to several different anti-cancer agents.

## Discussion

The ERK1/2 signaling pathway regulates various stimuli-induced cellular processes and plays a central role in human cancer^1–4^. Many inhibitors targeting this pathway have been approved or tested for cancer treatments^2,8,14–16^. Despite the success of several inhibitors, their efficacies are compromised by the emerging drug resistance or dose-limiting side effects^17–19^. Identification of more substrates regulated by this pathway may shed light on these problems. In the present study, we discovered that ERK1 and ERK2 are regulators of DHPS which is the key enzyme involved in hypusination. As Hypusination is a unique posttranslational modification that occurs on eukaryotic translational initiation factor eIF5A, which is required for mRNA shuttling, translational elongation, and termination^2,21^, our findings propose a new mechanism and function of ERK1/2 in regulating protein translation.

Our TAP-MS analysis revealed that DHPS interacts specifically and strongly with ERK1/2. We further validated that DHPS directly interacts with ERK1/2 via a conserved EBM at the N-terminus of DHPS, indicating that this interaction is evolutionally conserved. Furthermore, we found that DHPS is phosphorylated by ERK1/2 at Ser 233 and that this phosphorylation event is important for its optimal function in promoting cell proliferation. Thus, it is likely that ERK1/2 facilitates cell proliferation at least in part via its ability to phosphorylate DHPS and enhance hypusination and gene translation. In addition, DHPS expression strongly correlates with response to inhibitors of EGFR, MEK, and ERK1/2, which all act in the same pathway, suggesting that DHPS expression can be used as a predictive biomarker of therapy response.

Although our TAP-MS results suggested that DHPS is a major ERK1/2 binding protein, overexpression or knockdown of DHPS did not significantly affect ERK1/2 activation or activity (data not shown). On the other hand, DHPS is phosphorylated by ERK1/2 at the Ser-233 site. Moreover, we showed that DHPS phosphorylation increased after PMA treatment, which activates ERK1/2, suggesting that DHPS is regulated by the ERK1/2-dependent signaling pathway. Since the Ser-233 site is adjacent to the substrate spermidine binding groove^34^, we speculated that DHPS activity is regulated by ERK-dependent phosphorylation. Indeed, our follow-up experiments showed the decreased enzymatic activity of DHPS-S233A mutant. In addition, a study in β-cells associated DHPS/eIF5A hypusination with the activation of JNK and p38 pathway^39^, suggesting that DHPS may be regulated by multiple MAPKs, which need to be further investigated.

The augmented expression of DHPS was reported to correlate with poor prognosis in several tumor types, such as neuroblastoma and esophageal squamous cell carcinoma^29,40^. We found that higher expression of DHPS also correlated with poor survival in patients with lung adenocarcinoma. Moreover, we found that higher DHPS expression correlated with higher cellular resistance to inhibitors of EGFR pathway, such as several EGFR and MEK1/2 inhibitors, in lung cancer cell lines. These findings expand the possibility of using DHPS expression as a therapeutic biomarker in these types of cancers.

In summary, our findings of ERK1/2-mediated Ser-233 phosphorylation of DHPS not only expand our current understanding of the mechanism underlying the regulation of the hypusination pathway, but also provide new insights into the cellular processes affected by ERK1/2 pathway inhibition, which could be used as a therapeutic strategy for pathological conditions such as cancer.

## Methods and materials

### Cell culture

HEK293T and HEK293A cells were purchased from the American Type Culture Collection and cultured in Dulbecco modified essential medium supplemented with 10% fetal bovine serum at 37 °C in 5% CO_2_ (v/v). A549, H1666, H1792, H460, H1299, H1355, H1975, H23, H187 were purchased from the American Type Culture Collection and cultured in RPMI 1640 medium supplemented with 10% fetal bovine serum at 37 °C in 5% CO_2_ (v/v). The culture media contained 1% penicillin and streptomycin. All the cells were passed the test of mycoplasma. Plasmid transfection was performed with polyethyleneimine reagent, as reported previously^31^.

### Plasmids and chemical reagents

cDNAs of human DHPS, ERK1, and ERK2 were purchased from Addgene. All expression constructs were generated by polymerase chain reaction and subcloned into pDONOR201 vector as the entry clones using Gateway Technology (Invitrogen). All of the entry clones were subsequently recombined into a gateway-compatible destination vector to determine the expression of N-terminal/C-terminal SFB (S protein, Flag epitope, and streptavidin-binding peptide) or GST-tag fused proteins. DHPS mutation variants were generated via site-directed mutagenesis and then validated by Sanger sequencing. DHPS shRNAs were purchased from Dharmacon (shRNA1: RHS4430-200241186; shRNA2: RHS4430-200241004, shRNA3: RHS4430-200236611, and shRNA4: RHS4430-200233639).

### Tandem affinity purification of SFB-tagged proteins and MS analysis

HEK293T cells were transfected with plasmids encoding SFB-tagged proteins. Stable cell lines were selected with media containing 2 μg/ml puromycin and confirmed by immunostaining and Western blotting.

For TAP, HEK293T cells were subjected to lysis with NETN buffer (100 mM NaCl; 1 mM EDTA; 20 mM Tris HCl; and 0.5% NP-40) and protease inhibitors at 4 °C for 20 min. Crude lysates were subjected to centrifugation at 14,000 rpm for 20 min at 4 °C. The supernatant was incubated with streptavidin-conjugated beads (GE Healthcare) for 2 h at 4 °C. The beads were washed three times with NETN buffer, and bound proteins were eluted with NETN buffer containing 2 mg/ml biotin (Sigma-Aldrich) for 1 h at 4 °C. The elutes were incubated with S-protein agarose (Sigma-Aldrich) for 2 h, followed by three washes using NETN buffer. The beads were subjected to SDS-PAGE, and the gel was fixed and stained with Coomassie brilliant blue. The whole lane of the sample in the gel was excised and subjected to MS analysis. The MS analysis was performed as previously described^31^.

### Immunoprecipitation, western blotting, and antibodies

For pull-down assays, 1 × 10^7^ cells were lysed with NETN buffer containing protease inhibitors on ice for 20 min. Cell lysates were collected after centrifugation and incubated with 20 μL of S-beads for 2 h at 4 °C. The beads were washed with NETN buffer three times and boiled in 2× Laemmli buffer. The samples were resolved using SDS–polyacrylamide gel electrophoresis and transferred to a polyvinylidene fluoride membrane; immunoblotting was carried out with antibodies, as indicated in the figures.

Anti-ERK1/2 (9102, CST), anti-pERK1/2 (9101, CST), p-Ser/Thr (9631, CST) and anti-eIF5A (20765, CST) antibodies were purchased from Cell Signaling Technology and used at 1:1000 dilution. Anti-DHPS (NBP2-32256, Nova Biomedical) was used at 1:2000; anti-α-tubulin (T6199-200UL) and anti-Flag (M2) (F3165-5MG) monoclonal antibodies were purchased from Sigma-Aldrich and used at 1:5000 dilution. Anti-Hypusine antibody (ABS1064, Sigma) was used at 1:500 dilution.

### In vitro kinase assay

Recombinant DHPS-WT and DHPS variants were expressed in bacteria and purified using a GST protein tag. Both recombinant proteins were mixed with active ERK kinase (167820, United States Biological) and γ-32P-ATP (NEG002A100UC; PerkinElmer) in a kinase buffer (25 mM Tris [pH 7.5], 5 mM β-glycerophosphate, 2 mM dithiothreitol, 0.1 mM Na_3_VO_4_, and 10 mM MgCl_2_). These samples were incubated at 30 °C for 15 min. The reactions were stopped via boiling, and proteins were separated using sodium dodecyl sulfate-polyacrylamide gel electrophoresis. The gel was dried and imaged using the PhosphorImager software program.

### DHPS in vitro activity assay

The assay was performed as described previously^28^. In brief, the reaction mixture (25 μL) contained 0.4 mM NAD, 1 mg/mL BSA, 1 μg of GST-eIF5A, 2 μCi of [1,8-terminal methylenes-3H] spermidine (PerkinElmer/NEN), and 1 mM DTT in 0.2 M glycine NaOH buffer (pH 9.5) with the indicated amounts of 0.2 μg purified GST-DHPS variants. After incubation at 37 °C for 1.5 h, 0.5 mg of BSA (bovine serum albumin) protein was added and the reaction was immediately stopped by the addition of 1 mL of 10% TCA (trichloroacetic acid) containing three polyamines (Sigma-Aldrich); putrescine (1 mM), spermidine (1 mM), and spermine (1 mM). The TCA-precipitated proteins were collected by centrifugation at 15,000 × *g* in a refrigerated microcentrifuge for 5 min. After removal of supernatants containing unreacted radioactive spermidine, the precipitates were re-suspended in a 10% TCA solution containing polyamines. The wash was repeated twice to remove all of the unreacted radioactive spermidine. The TCA-washed protein precipitates were then dissolved in 0.1 mL of 0.15 M NaOH, and the radioactivity incorporated into eIF5A protein was measured using a PerkinElmer liquid scintillation counter. The enzyme activity was calculated after subtraction of the background radioactivity bound to TCA precipitates of the reaction mixture lacking any enzyme.

### RNA isolation and quantitative real-time PCR

Total RNA was prepared using TRIzol (Invitrogen) and reverse transcribed using the PrimeScript RT Reagent Kit with genomic DNA Eraser (Takara, RR047A). The qPCR reactions were run in an ABI Q6 real-time PCR instrument. Levels of DHPS mRNA were detected by the TaqMan MicroRNA assay (ABI Scientific) and normalized by β-actin mRNA. The primers used in this study were as follows: (β-actin forward: CACCATTGGCAATGAGCGGTTC; β-actin reverse: AGGTCTTTGCGGATGTCCACGT; DHPS forward: GTTCAGGCATCCGTGAGACCAT; DHPS reverse: CAGGCACTTGATGAGGTCTTCC).

### Cell growth and clonogenic survival assays

To determine the cell growth curve, we plated 2 × 10^5^ cells in 60 mm dishes and counted them daily. Media were changed every day.

Clonogenic survival assays were performed as described in a previous paper (28). In brief, 250 indicated cells were seeded onto 6-well plates, and the cells were incubated for 12 days. The colonies were stained with crystal violet and counted manually. The results were the averages of data from three independent experiments, and the statistical analysis was performed using Student’s *t* test.

### Kaplan–meier plot and drug sensitivity correlation

A Kaplan–Meier plot was generated with a Kaplan–Meier plotter, as described by Nagy et al.^41^. The DHPS probe 211558_s_at was used. 865 patients were included.

The drug sensitivity correlation datasets were derived from the Genomics of Drug Sensitivity in Cancer project, and CellMinerCDB was used to analyze the data, as described in a previous paper^37^. The –log10IC50M were used to mark the drug activity. Pearson correlation was used to evaluate the correlation between gene expression levels and drug activities.

### Quantification and statistical analysis

The Western blot signals were quantified with software ImageJ (National Cancer Institute).

Each experiment was repeated three or more times unless otherwise noted. Differences between groups were analyzed using Student’s *t* test, one sample *t*-test or two-way analysis of variance with the Tukey multiple comparisons test. *P* values < 0.05 were considered statistically significant.

## Supplementary information

Supplemental Figures and Figure Legends

Supplementary Table 1

Supplementary Table 2

Change of author/authorship agreement
