# Supplementary material for: Extracellular signal-regulated kinases associate with and phosphorylate DHPS to promote cell proliferation
Source: Oncogenesis. 2020 Sep 28;9(9):85. doi: 10.1038/s41389-020-00271-1 (PMC7522278; doi:10.1038/s41389-020-00271-1)
Supplement: Supplementary file 1 — Supplemental Figures and Figure Legends [file 41389_2020_271_MOESM1_ESM.pdf]

SUPPLEMENTAL INFORMATION

SUPPLEMENTARY FIGURES AND FIGURE LEGENDS

A

N-ERK2

Gene	Unipep	Pep	PSM
MAPK1	26	36	839
MAPK3	1	11	218
MAPK7	29	29	151
RPS6KA1	22	32	78
DHPS	16	16	53
RPS6KA3	14	25	63
HIST1H1E	2	12	20
HIST1H2BD	2	7	24
HIST1H2BO	2	7	24
RPS6KA2	3	9	28
STOML2	13	13	27
NLK	1	2	14

C-ERK2

Gene	Unipep	Pep	PSM
MAPK1	28	38	1400
MAPK3	3	13	405
RPS6KA1	42	65	362
RPS6KA3	35	59	298
DHPS	23	23	156
RPS6KA2	16	33	122
RPS6KA6	9	22	64
DUSP9	15	17	45
HIST1H1E	3	14	24
HIST1H2BD	2	8	22
HIST3H2BB	3	8	20
FLT3	1	2	20
NLK	2	3	22

B

N-DHPS

Gene	Unipep	Pep	PSM
DHPS	26	26	672
MAPK1	16	19	96
MAPK3	12	15	69
HSPA1A	15	18	50
HSPA8	17	20	49
ACACA	26	26	38
KRT1	13	17	36
EIF5A	6	6	26
MMP8	1	1	21
UBC	3	3	18
SLC25A5	4	10	18

C-DHPS

Gene	Unipep	Pep	PSM
DHPS	33	33	1015
KRT1	30	33	92
EIF5A	10	10	80
HSPA8	19	21	57
UBC	8	8	34
SLC25A5	3	8	34
MAPK3	9	9	19
SLC25A6	1	6	14
MAPK1	6	6	11
MMP8	1	1	7

C

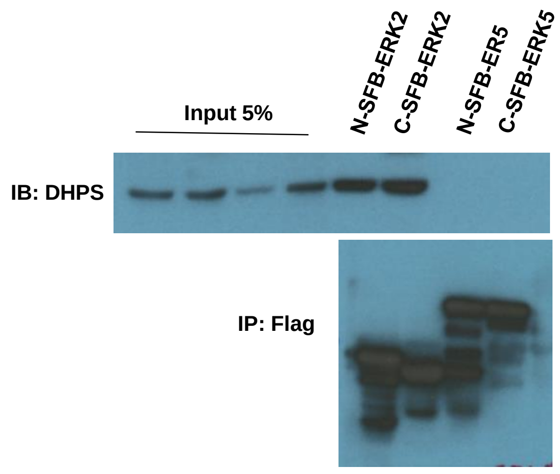

D

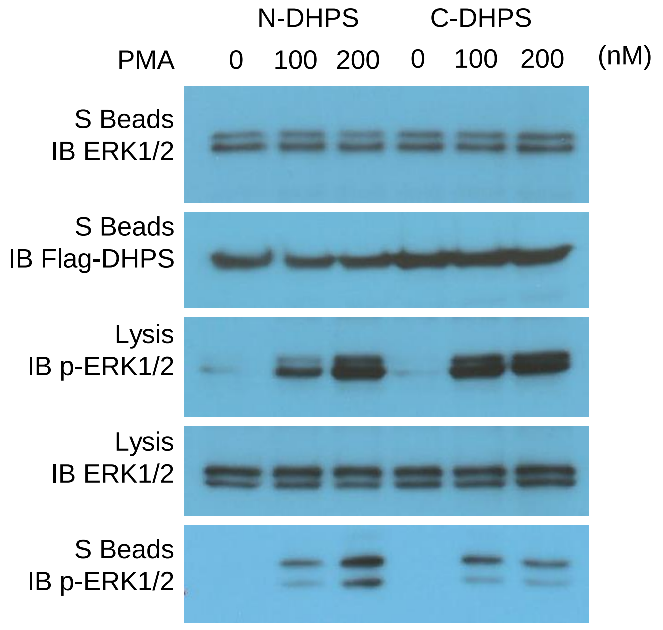

Supplementary Figure 1 (Related to Figure 1)

(A) The HCIP list resulting from TAP-MS analysis of N- and C-terminal SFB-tagged ERK2. The bait and reported interactors are marked with green. The candidate proteins are marked with red.

(B) HCIP list resulting from a TAP-MS analysis of N- and C- terminal SFB-tagged DHPS. The bait and reported interactors are marked with green. The candidate proteins are marked with red.

(C) ERK2 but not ERK5 interacts with DHPS. N- or C-terminal SFB-tagged ERK2 or ERK5 were expressed in HEK293T cells. Cell lysates were pulled down with S protein beads, and the indicated proteins were detected by Western blotting.

(D) Association between DHPS and ERK1/2. N- or C- terminal SFB-tagged DHPS was expressed in HEK293T cells. Cell were treated with DMSO or different dosages of PMA for 30 minutes. Cell lysates were pulled down with S protein beads, and the indicated proteins were detected by Western blotting.

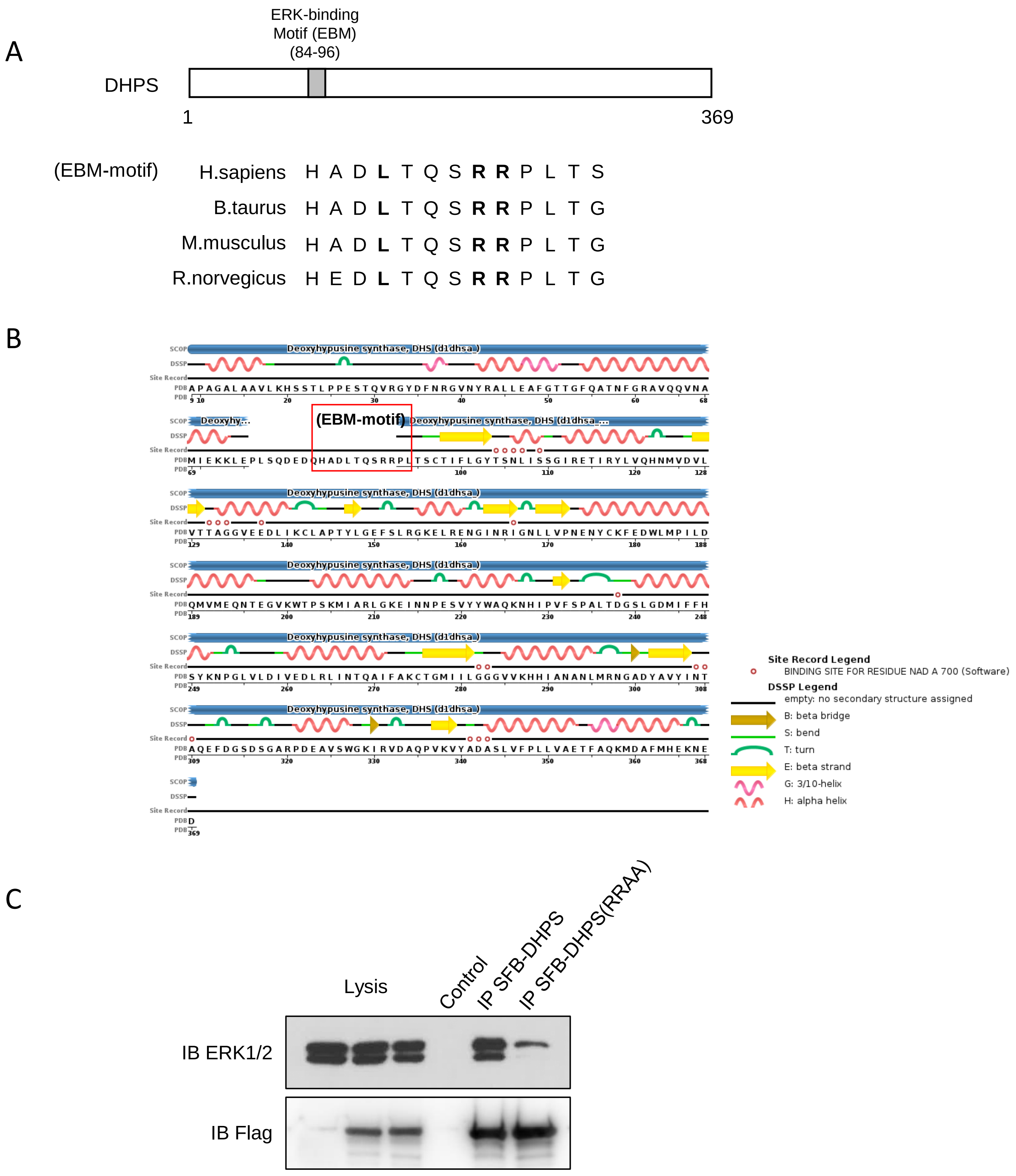

Supplementary Figure 2. (Related to Figure 2)

(A) Sequence alignment of EBM between DHPS from humans and other species.

(B) Secondary structure of DHPS protein. EBM sequence is marked by a red rectangle.

(C) Mutations of negatively charged residues in the EBM of DHPS dramatically decrease the binding between DHPS and ERK1/2. SFB-tagged WT or the RRAA variant of DHPS was expressed in HEK293T cells. Cell lysates were pulled down with S protein beads, and the indicated protein was detected by Western blotting. Data represent a representative experiment from three independent experiments.

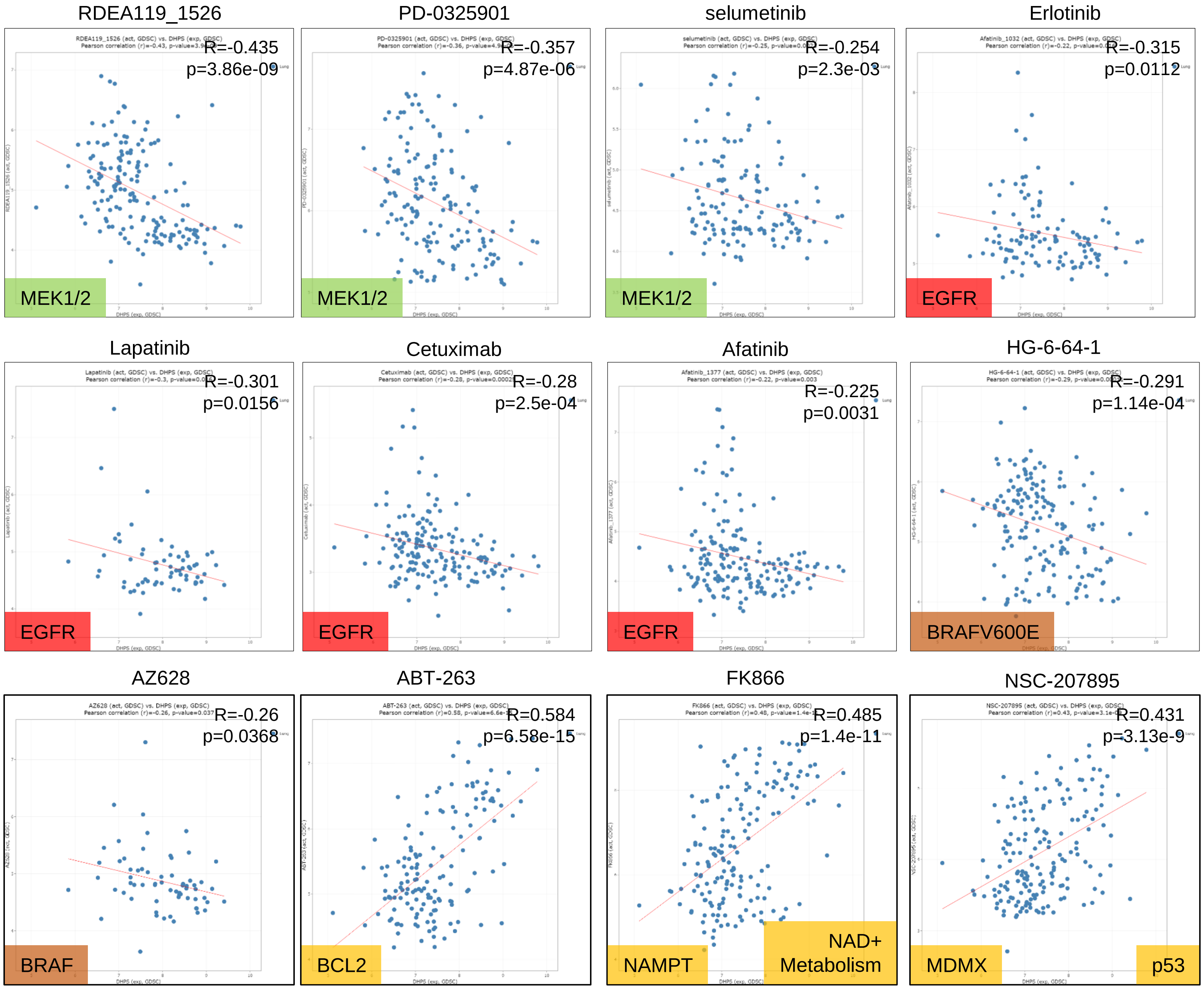

Supplementary Figure 3. (Related to Figure 5)

The top negative and positive correlated drugs from Figure 5 (B) were plotted. The Pearson correlation R and the p values are labeled in the panels.

SUPPLEMENTARY TABLE CAPTIONS

Supplementary Table 1 (Related to Figure 1). TAP-MS lists of DHPS and ERK2

Supplementary Table 2 (Related to Figure 5). Correlation between Drug activities and DHPS mRNA expression levels
