# Supplementary material for: Extracellular signal-regulated kinases associate with and phosphorylate DHPS to promote cell proliferation
Source: Oncogenesis. 2020 Sep 28;9(9):85. doi: 10.1038/s41389-020-00271-1 (PMC7522278; doi:10.1038/s41389-020-00271-1)
Supplement: Supplementary file 4 — Change of author/authorship agreement [file 41389_2020_271_MOESM4_ESM.pdf]

SPRINGER NATURE

In accordance with Springer Nature Authorship Policy we agree to change the authors of the manuscript as indicated below.

NAME OF JOURNAL: Oncogenesis

TITLE OF MANUSCRIPT: Extracellular Signal-Regulated Kinases Associate with and Phosphorylate DHPS to Promote Cell Proliferation

MANUSCRIPT NUMBER: ONCSIS-20-0357R

CORRESPONDING AUTHORS NAME: Junjie Chen

PREVIOUS AUTHOR NAMES:

Chao Wang , Zhen Chen , Litong Nie , Mengfan Tang , Xu Feng , Dan Su , Huimin Zhang , Yun Xiong

UPDATED AUTHOR NAMES:

Chao Wang , Zhen Chen , Litong Nie , Mengfan Tang , Xu Feng , Dan Su , Huimin Zhang , Yun Xiong , Joeng-Min Park

CHANGE TO AUTHOR LIST:

Joeng-Min Park

Print Name	Signature	Date
Junjie Chen	[signature]	08/07/2020
Chao Wang	Chao	08/07/2020
Zhen Chen	Zhen Chen	08/07/2020
Litong Nie	li Tong	08/07/2020
Mengfan Tang	Tang mengfan	08/07/2020
Xu Feng	Xu feng	08/07/2020
Dan Su	DAN	08/07/2020
Huimin Zhang	Huimin	08/07/2020
Yun Xiong	Xiong Yun	08/07/2020
Joeng-Min Park	Joeng Min Park	08/07/2020
